# Challenges and realities of early childhood development centers in Malawi: A critical examination

**DOI:** 10.1371/journal.pone.0314530

**Published:** 2025-02-21

**Authors:** Lazarus Obed Livingstone Banda, Chigonjetso Victoria Banda, Jane Thokozani Banda

**Affiliations:** 1 Computer Studies Department, Nalikule College of Education, Ministry of Education, Lilongwe, Malawi; 2 Department of Politics and Government, School of Law, Economics and Government, University of Malawi, Zomba, Malawi; 3 Directorate of Higher Education, Ministry of Education, Lilongwe, Malawi; Makerere University / Mulago National Referral Hospital, UGANDA

## Abstract

The study on Early Childhood Development (ECD) practices in T/A Zilakoma, Nkhata Bay South Constituency, Malawi, employs the Ecological Systems Theory to explore recruitment, role definitions, and support systems. This theoretical construct enables an intricate examination of interactions within various environmental systems, emphasizing micro, meso, exo, macro, and chrono systems. Specifically, it illuminates the dynamics within immediate settings, interconnections among diverse systems, broader indirect influences, cultural ideologies, societal values, and temporal dimensions, offering a comprehensive lens for understanding educational contexts. The descriptive qualitative research was conducted within a case study framework to explore the practical experiences of stakeholders within ECD using semi-structured interview guides. Ethical standards were upheld, ensuring voluntary participation and confidentiality. Purposive sampling was used to collect data from diverse and knowledgeable participants involved in ECD domains, providing comprehensive insights aligned with the study’s objectives. Thematic analysis and sentiment mining were performed using Atlas 23 software. The results revealed themes such as recruitment practices relying on community-driven approaches, role ambiguity due to undefined responsibilities, informal evaluation processes, inconsistent training opportunities, and a dependency on community and volunteerism. These themes highlight the absence of formal structures and standardized processes in various aspects of ECD programs. Additionally, sentiment analysis illustrated diverse perspectives among stakeholders, reflecting their distinct experiences and challenges within the ECD landscape. The study concludes with policy recommendations aimed at addressing these systemic challenges.

## Introduction

Early Childhood Development (ECD) is widely acknowledged as a critical foundation for long-term academic and social success, particularly in resource-constrained settings. In Malawi, the importance of ECD is recognized in the country’s legal frameworks, which aim to support holistic child development. However, despite such frameworks, ECD in Malawi remains hindered by a range of systemic and resource-related challenges, especially within rural Community-Based Childcare Centres (CBCCs). These centers, often reliant on volunteer-based staffing, struggle with unstructured recruitment processes, unclear role definitions, and limited professional support for educators. Such challenges undermine the effectiveness of ECD services, particularly in rural areas where access to early learning opportunities is already scarce.

This study seeks to explore these challenges in depth, focusing on the recruitment, role clarity, and support mechanisms of ECD teachers in Malawi’s rural CBCCs. Specifically, it aims to examine the recruitment practices and their impact on ECD outcomes, clarify the roles and responsibilities of ECD teachers, and assess the support structures in place for their professional development. Additionally, the study seeks to gather insights from key stakeholders—ECD teachers, community members, welfare department officers, and Ministry of Education officials—to understand their experiences and perceptions of the current system. By analyzing the existing practices within these centers, the study aims to identify the factors that influence their effectiveness and propose strategies to enhance ECD outcomes in the region.

Despite growing attention to the importance of ECD, significant research gaps persist, particularly regarding the recruitment and retention of qualified educators in Malawi’s rural areas. While previous studies have focused on challenges such as inadequate infrastructure and resource limitations, little is known about how recruitment practices and the lack of clear job descriptions impact the quality of education provided. Moreover, the need for systematic research on the professional development and support mechanisms further hinders the potential for improving ECD services. Addressing these gaps is essential for developing a more comprehensive understanding of the barriers to effective ECD implementation in Malawi.

This study is relevant not only in the context of Malawi but also more broadly for developing countries facing similar challenges in delivering quality ECD services. By providing actionable insights into recruitment, role clarity, and support mechanisms, the findings contribute to the global discourse on achieving the Sustainable Development Goals (SDGs) related to education and child welfare. Furthermore, the study’s implications for policy are significant. It highlights the need for more structured and formalized recruitment processes, clearer role definitions, and the establishment of continuous professional development opportunities for ECD educators. These recommendations, if implemented, could strengthen the overall ECD system in Malawi, improve educational outcomes for young children, and serve as a model for other countries facing similar challenges in the Global South.

## Literature review

The importance of ECD in fostering long-term academic and social success is well documented. However, its integration into mainstream education systems is often hampered by inadequate infrastructure, insufficient funding, and a lack of trained educators as seen in Zimbabwe and Ethiopia [1–3] which are critical for fostering an inclusive environment for all children, especially those with learning barriers [4–6]. In Africa, China, and Europe, poverty and socio-economic inequality limit access to quality early childhood education among children from disadvantaged backgrounds [2,7–10].

Like Europe, Latin America grapples with Europe’s fragmented and random policy implementation, inconsistencies between policy and practice, and limited coordination among stakeholders emanating from political issues hamper efforts to streamline ECD initiatives [2,10–12]. These challenges further complicate the ability to provide quality education across all regions [8,12–14]. Despite job description and division of labor being critical for efficiency [15], many stakeholders lack coordinated approach to ECD leading to unequal and uneven implementation [13].

In developing countries, there is a lack of supportive physical and social environment and poor and inadequate infrastructure [16,17]. The region also faces challenges related to the discrimination against children with disabilities [18] reports on the barriers these children face in accessing inclusive ECD programs across South Asia [19]. These challenges are compounded by a lack of infrastructure and trained personnel to support children with special needs, leading to unequal educational outcomes for vulnerable populations.

Despite research indicating the importance of well-trained ECD teachers and caregivers [20], the Global South still grapples with understaffing and unskilled human resource [3,11,16] and insufficient and outdated pedagogical resources [5,11]. High teacher-pupil ratios lead to overcrowded classrooms, evident in Namibia [21] and Zimbabwe [1], and hinder effective learning. Additionally, Lassalle points to the inadequate professional development opportunities for teachers as a significant hindrance to improving the quality of early childhood education in the Latin American region [12]. Untrained volunteers in ECD teaching can yield both positive and negative impacts on educational outcomes.

While their enthusiasm and fresh perspectives can enhance classroom dynamics, their lack of or inconsistent formal training [2] often results in gaps in understanding and implementing effective educational practices. This is particularly evident in play-based learning, where untrained practitioners may struggle to fully appreciate the complexity of children’s learning experiences through play, as demonstrated in Malaysian ECE centers [22]. Volunteers frequently lack a deep understanding of the educational value of play, which is crucial for children’s social and cognitive development. In Malaysia, for example, untrained ECE practitioners initially failed to recognize the learning opportunities inherent in children’s play, such as sharing resources or understanding peers’ emotions [22]. Without this critical awareness, volunteers may inadvertently overlook key developmental milestones, reducing the effectiveness of play-based learning. The lack of trained educators also impacts the broader implementation of ECD programs. In Zimbabwe and Ethiopia, the absence of trained ECD teachers and play centers has been recognized as a barrier to the effective implementation of ECD programs [3,23].

Similarly, the Sub-Saharan Region, Latin America, and Europe also struggle with curriculum-related issues [3,8,10–12,24–26]; For instance, the use of non-native languages in instruction often alienates children from diverse linguistic backgrounds, limiting their ability to engage in the learning process fully [21]. Unprocedural teacher recruitment and volunteering services without training in ECD significantly affect the quality of education [3]. In Europe, there is a worrisome gender imbalance among ECD caregivers and teachers [10]. In Kenya, decentralized recruitment processes have strongly correlated with educational outcomes, with recruitment practices demonstrating a significant statistical relationship with ECD quality [27]. Despite this, global challenges persist in recruiting and retaining qualified ECD teachers due to low wages, inadequate professional development, and poor working conditions [28]. These issues are further compounded by the marginalization of the early childhood profession, which complicates policy efforts to increase the teacher supply [29].

While unprocedural recruitment poses significant challenges, addressing fundamental issues such as professional recognition, wages, and working conditions could enhance recruitment outcomes. A holistic ecological perspective on workforce issues may offer sustainable solutions for attracting and retaining ECD teachers [29]. In all these regions, the common thread of resource constraints, lack of qualified educators, and inconsistent policy implementation persists [11], highlighting the global nature of ECD challenges. Whether through socio-economic barriers, infrastructural deficits, or inadequate support for educators, these challenges collectively hinder providing equitable and high-quality early childhood education across different contexts. Studies from various regions underscore the significance of Holistic Child Development and the need for systematic approaches to address these challenges [30,31].

The comprehensive exploration into ECD practices, encompassing recruitment, role delineation, and support systems, finds resonance within the theoretical framework of the Ecological Systems Theory (EST) posited by Urie Bronfenbrenner [32–35]. This theoretical construct offers a sophisticated lens to discern the intricate dynamics governing human development within various environmental systems. At its core, this theory delineates a hierarchical model encompassing the microsystem, mesosystem, exosystem, macrosystem, and chronosystem, elucidating the multifaceted interactions prevalent within educational contexts [36].The microsystem, constituting the immediate environments of direct interaction, manifests prominently within this study, encapsulating the daily interactions among ECD teachers, community members, and young learners within educational settings. Here, the EST allows an intricate examination of how roles, recruitment methodologies, and support mechanisms operate within these micro-contexts. Furthermore, the mesosystem, focusing on interconnections between diverse microsystems, elucidates the complex interfaces between ECD teachers, community recommendations, welfare departments, and educational authorities, unraveling the intricate interplays and influences shaping early childhood education.

EST captures the broader, indirect settings influencing individuals’ experiences by expanding the purview to the exosystem. Within the study, this tier encompasses the broader community norms, societal expectations, and governmental policies shaping recruitment practices, evaluation methodologies, and support structures within the realm of ECD. Moreover, the macrosystem, entrenched within overarching cultural ideologies and societal values, illuminates how societal perceptions of education, community involvement, and volunteerism intricately mold observed practices within early childhood education [35]. Complementing these tiers, the chronosystem integrates temporal dimensions, tracing developmental trajectories and temporal changes. The study’s context encapsulates the evolving socio-economic factors, educational policies, and resource availabilities, unveiling the temporal evolution of recruitment, roles, and support mechanisms within ECD programs. Thus, adopting EST within this study offers a nuanced, multi-tiered framework to unravel the intricate interdependencies, multifaceted interactions, and evolving dynamics among stakeholders entrenched within early childhood education contexts.

## Methods and materials

### Design

This study employs a descriptive qualitative research design within the framework of an embedded case study, focusing on ECD challenges within T/A Zilakoma, Nkhata Bay South Constituency. The case study design enables an in-depth exploration of ECD practices across multiple schools within the region, providing rich insights into the localized challenges that teachers and stakeholders face. Semi-structured interviews were conducted with ECD teachers from six schools, community members, and officials to capture their perspectives and experiences. Thematic analysis was used to identify recurring patterns and themes across these embedded units, offering a comprehensive description of the ECD landscape within this case. The study design ensured meticulous attention to ethical considerations, adhering to protocols and guidelines for research involving human participants. Informed consent was obtained from all participants, ensuring voluntary participation and safeguarding their confidentiality and anonymity throughout the research process.

### Sample size and description

There were 25 respondents in this study. Table 1 is a demographic summary of the sample.

**Table 1:** A demographic summary of the sample.

Participant Category	N	Female	Male	Mean Age	Work Experience	Educational Background	Trained in ECD	Location
**ECD Teachers**	15	15	0	28	1	10 Junior High School 5 Senior High School	None	Rural
**Community Members**	5	2	3	40	N/A	2 Primary 3 Secondary	None	Rural
**Welfare Department Officers**	3	1	2	45	7	1 Diploma 1 Bachelor’s 1 master’s	1 trained 2 untrained	Mixed
**Ministry of Education Officials**	2	1	1	50	13	1 Bachelor’s & 1 Master’s	Not trained	Urban

The table illustrates that all the teachers/caregivers were females and had worked for an average of one year.

### Sampling technique

The study employed purposive sampling to select participants with relevant and diverse experiences in ECD, ensuring a comprehensive understanding of the ECD landscape. Participants were drawn from key groups, including ECD teachers, community members, welfare department officers, and Ministry of Education officials, chosen for their direct involvement in ECD activities and insights into the field.

Diversity in demographic background, professional roles, and geographic location was prioritized to capture various perspectives. Participants were selected based on their active engagement in ECD, with an emphasis on those possessing substantial experiential knowledge relevant to the study’s objectives. This included both frontline educators and administrative officials, allowing for exploring practical and policy-related insights. This method aligned with the research objectives, aiming to capture multifaceted experiences related to ECD recruitment, roles, and support mechanisms.

Additionally, efforts were made to ensure gender balance across the sample, ensuring equal representation of male and female participants in each group, except among the teachers/caregivers, who were all females in all the six ECD centers involved.

### Data collection

The data collection instruments for this study were carefully designed to capture in-depth insights into ECD practices. A structured in-depth interview guide and an observation checklist were developed and tested for validity through a pilot study, ensuring they effectively aligned with the research objectives. Additionally, field notes were used during both interviews and observations to document non-verbal cues, environmental context, and other relevant details that may not have been captured by audio recordings alone.

Two high school teachers, who were native speakers of Tonga, were specially trained as professional enumerators. Their training included cultural sensitivity to ensure smooth communication and accurate interpretation of both verbal and non-verbal cues, allowing for appropriate coding and decoding of participant responses. In addition, the study incorporated two natives from the catchment area who were fluent in the local dialects. This not only facilitated a deeper understanding during data collection but also fostered a greater sense of identity and trust between the respondents and enumerators, enhancing the overall quality of the responses.

Data collection took place over a four-month period, from July 3rd, 2022, to October 28th, 2022, following ethical clearance obtained in June 2022. Interviews were conducted using the in-depth interview guide, and observations were made using the checklist, with the enumerators documenting both verbal responses and additional field notes. All interviews were audio-recorded with participants’ consent, ensuring accuracy while maintaining confidentiality.

The sample size was determined by data saturation among the ECD teachers, ensuring that the point at which no new information was emerging had been reached. This approach provided comprehensive and representative insights into the varied experiences within the ECD landscape. The collected data underwent systematic thematic analysis to identify key patterns and themes, contributing valuable insights into ECD practices and challenges in the region.

During the data collection process, several challenges emerged that required mitigation strategies to ensure successful data gathering. Key challenges included potential biases in participant responses, logistical constraints, and the need to create a conducive interview environment.

Mitigating biases was essential to ensure data authenticity. The trained enumerators adopted neutral stances during interviews, fostering an open and non-judgmental atmosphere that encouraged honest responses. Building rapport with participants before the interviews further enhanced trust and reduced potential biases in their responses.

Logistical constraints, including travel and scheduling conflicts, were managed through careful planning and flexibility. The researchers worked around participants’ schedules and often conducted interviews at convenient times and locations, ensuring full participation.

Creating a conducive environment for interviews was crucial for obtaining candid responses. Privacy and confidentiality were prioritized, with interviews held in comfortable and neutral locations to make participants feel at ease. Ethical standards were strictly followed, with oral informed consent obtained from all participants and recorded using voice recorders with their full knowledge. For minors observed during “classes,” caregivers or teachers provided oral consent, which was similarly documented.

### Data analysis

The methodological approach adopted for this study involved a systematic process encompassing several interconnected stages to derive a comprehensive thematic analysis from the interview responses. Audio data were transcribed into written form, allowing for a comprehensive review and understanding of the content.

We recruited two professional transcribers who did not know each other and vetted each other’s work for accuracy. However, they were informed of the “collaboration.” Following transcription, the process involved familiarization with the data, whereby a thorough review of the responses was conducted to gain a nuanced understanding of the information provided. The transcripts were translated into English, a step pivotal in preparing for the subsequent phases of analysis.

Data was then imported into Atlas 23 for a thematic analysis in which all the documents were imported. The approach pertains to discerning, scrutinizing, categorizing, explicating, and documenting recurring patterns and concepts in our data. The thematic analysis was an intermediary for us, employing distinct qualitative and quantitative analytic approaches facilitating effective communication and understanding between them [37]. The chosen methodology was based on recognizing that a thorough theme analysis could yield reliable and profound discoveries [37]. Besides, this versatile methodological approach was utilized to find, analyze, and interpret recurring themes or patterns within our datasets to gain a comprehensive knowledge of the data presented in the interviews, as the methodology enabled insights into individuals’ experiences, attitudes, and behaviors on a comprehensive scale. Utilizing a subjective method enabled the establishment of connections between several hypotheses [38,39]. Subsequent to familiarization, an initial coding process was initiated, involving identifying recurring themes or patterns present within the responses. Codes were assigned to specific excerpts or quotes that pertained to similar thematic areas, facilitating the organization and grouping of related content.

The data were further analyzed to develop preliminary themes after the initial coding phase. This process involved collating the coded excerpts into broader thematic categories based on the identified patterns. An iterative approach was adopted to review and refine these themes, ensuring the comprehensive coverage and accuracy of the analysis. Additionally, cross-validation was conducted to check for consistency and coherence of themes across different participant groups, validating the robustness of the identified themes.

Throughout the analytical process, representative quotations from each participant group were integrated under respective thematic categories to elaborate and support the identified themes. This step was crucial in providing illustrative evidence and grounding the analysis within the context of the interviews. Besides, sentiment mining helped interpret the data more [40,41]. The finalized analysis was structured cohesively and coherently, ensuring a detailed representation of findings to present a comprehensive understanding of the data derived from the interviews.

### Ethical clearance

This study was cleared by the Mzuzu University Research Ethics Committee under the project of “Performance in the Malawian Education System: Who is the smartest,” with approval reference number Ref No: MZUNIREC/DOR/21/55.

## Results

**Fig 1:**
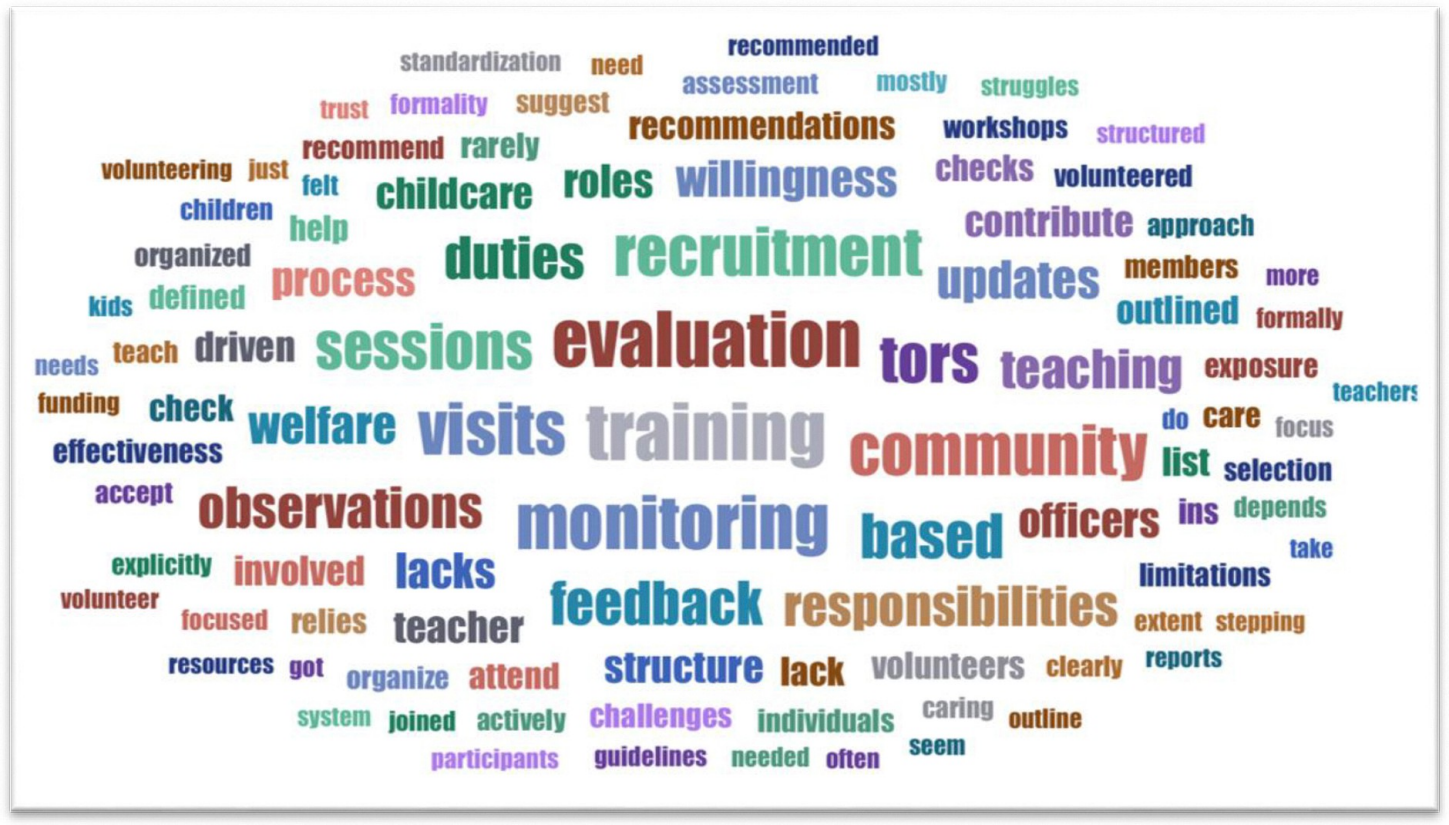
Word cloud indicating some of the keywords in developing themes.

The thematic analysis delves into the perspectives of ECD teachers, community members, welfare department officers, and Ministry of Education officials regarding recruitment, roles, and support mechanisms within the early childhood education domain. The word cloud in Fig 1 guided the development of themes in line with the developed codes.

### Theme 1: recruitment practices

#### Volunteering, career path, and turnover

This theme reveals the absence of formal recruitment structures in ECD centers, where recruitment predominantly relies on community recommendations and volunteerism. Participants consistently highlighted that social trust and personal connections drive recruitment, rather than standardized procedures, leading to inconsistencies in the selection of teaching staff and volunteers. This reliance on informal practices has significant implications for the quality and sustainability of ECD programs.

You quickly volunteer once the person requesting you or among the community group is your bestie or someone you respect, or if you have nothing to do. It’s a way to pass your hours away. But then, the zeal soon wanes away.–Community Member 2

Most volunteers give up before they’ve even completed a year. It’s hard to keep them when there’s no proper pay or job security.–Community Member 2

This quote illustrates the frustrations faced by volunteers, who often take on teaching roles without clear expectations or long-term prospects. Volunteers struggle with minimal compensation and lack of financial support, making it difficult for them to justify their continued involvement. As a result, high turnover rates destabilize the learning environment, leading to further challenges in maintaining consistency in ECD services.

From the perspective of the volunteers, the lack of professional development and growth opportunities is a key reason for their early departure. Many volunteers expressed frustration with the absence of a clear career path, them feeling undervalued and unmotivated to continue in their roles.

There’s no real future in it. You’re just helping for now, but it doesn’t lead to anything more.–ECD Teacher 5

This sentiment reflects a critical issue: volunteers do not view ECD work as a long-term career option due to the absence of structured professional advancement opportunities. Without clear pathways for growth or sufficient monetary incentives, it becomes increasingly difficult for volunteers to remain committed. The high turnover of volunteers, the absence of welfare officers during the recruitment process, and the reliance on community-driven approaches collectively contribute to a fragmented recruitment system that struggles to maintain continuity and quality in the ECD sector. Volunteers often leave due to lack of compensation, career prospects, and formal support structures, while the failure of welfare officers to engage at critical stages of recruitment exacerbates these challenges. Without formal interventions or structured recruitment processes, the cycle of instability within the ECD workforce is likely to persist, undermining the effectiveness and sustainability of early childhood education programs.

### Limited oversight in recruitment and service quality

When asked whether welfare officers are involved in the recruitment of caregivers, participants consistently indicated that there is little to no oversight from these officials. Teachers and community members pointed out that welfare officers rarely engage in the recruitment process, leaving this responsibility largely in the hands of local communities, without formal guidance or oversight.

The welfare officers don’t really get involved when we recruit caregivers. It’s more of a community-driven process, and they’re not around to give us any support.–Welfare Department Officer 2

This quote underscores the informal nature of recruitment practices, with minimal intervention or oversight from welfare officers. The lack of a formalized recruitment structure perpetuates the reliance on volunteerism and community recommendations, with no standardized guidelines in place to ensure the quality of teachers being recruited. This reliance on informal practices creates a fragmented recruitment system that struggles to maintain quality and consistency in ECD staffing.

In fact, there is no formal structure, and it is often community-driven.–Ministry of Education Officer 1

This sentiment from a Ministry of Education official highlights the broader systemic issue where the absence of formal recruitment processes is accepted as the norm. Without institutionalized standards, the quality and consistency of recruitment are compromised, creating a significant risk to the overall effectiveness of the ECD programs.

The absence of a formal recruitment structure raises concerns about the consistency and fairness of the recruitment process. Without clear guidelines or standardized criteria, there may be a disconnect between the qualifications or capabilities of those recruited and the needs of the educational programs they are expected to support. This lack of structure hinders the development of a professional teaching workforce and limits the effectiveness of ECD initiatives.

Field notes and observations supported these findings, as no welfare officers were observed participating in recruitment-related activities at any of the schools visited. This lack of oversight and structure leads to inefficiencies in ensuring that those recruited have the necessary skills or training for their roles, potentially impacting the overall quality of ECD services.

### Theme 2: Role ambiguity

The theme of role ambiguity reveals the lack of formal role definitions for teachers, where the terms of reference (TORs) are loosely defined and duties remain unspecified. Interviews consistently indicated that, rather than operating within a structured framework, teachers often take on flexible and informal roles, shaped by immediate needs and their willingness to help. This absence of clear role definitions leads to disorganized and inconsistent practices, with teachers left to determine their responsibilities without guidance.

There’s no clear outline; we just do what we can to teach and care for the children.–ECD Teacher

This statement underscores the lack of formal role definitions, with teachers engaging in activities based on what they can manage rather than on predetermined tasks or expectations. The participant’s remark suggests that this flexibility can lead to varying approaches in teaching and caregiving, ultimately resulting in inconsistencies in the quality of services provided.

Teachers take care of kids without a formal list of duties.–Community Member 1

A community member’s observation supports the theme, noting that teachers operate without specific guidelines or responsibilities. The lack of a formal duty list creates confusion about what is expected of ECD teachers, potentially leading to unequal task distribution. As a result, some teachers may take on more responsibilities while others may do less, further contributing to operational disorganization.

- “No defined TORs, responsibilities based on willingness.”–Welfare Department Officer 1

The welfare department officer emphasized that teachers’ roles are often determined by their personal willingness rather than a formal assignment of duties. This raises concerns about accountability and efficiency, as teachers may not fully understand their professional responsibilities and may prioritize tasks based on preference rather than institutional needs.

Roles seem flexible, not outlined formally.–Ministry of Education Officer 1

A ministry official also noted the flexibility in roles, acknowledging that while adaptability might be beneficial in certain situations, the absence of formal structure compromises organization and the effective delivery of educational services. Without formal guidelines, it becomes challenging to ensure that all aspects of teaching and childcare are addressed in a cohesive manner.

Field observations and notes corroborated the thematic analysis on role ambiguity. Teachers were observed performing multiple unrelated tasks—ranging from administrative duties to caregiving—without clear distinctions between roles. This lack of formal structure in daily operations was evident across various settings. The reliance on volunteerism and the absence of formal training further mirrored the participants’ accounts of inadequate support systems, validating the challenges related to role ambiguity and informal recruitment practices.

The consistency between the observational data and the interview insights strengthens the overall analysis. It provides a comprehensive understanding of how role ambiguity and undefined responsibilities negatively affect the operational effectiveness of ECD programs, reinforcing the need for clear role definitions and structured support systems to enhance teacher efficiency and program quality.

#### Impact of undefined responsibilities on ECD operations

The lack of clearly defined TORs results in disorganized operations and risks creating confusion about the scope of teachers’ responsibilities. Teachers are left to determine their roles based on personal judgment, which can lead to inefficiencies in task execution and unequal task distribution. Additionally, this lack of structure places an extra burden on teachers to self-manage, leading to potential burnout or reduced effectiveness over time. The absence of formal role definitions also compromises the ability of the program to run efficiently, as teachers may prioritize tasks based on individual preferences rather than the needs of the program.

A more structured and clearly defined set of responsibilities would help create an organized and efficient system, ensuring that teachers are fully aware of their roles and can collaborate effectively to meet the program’s goals. This would improve the consistency of service delivery and the overall quality of the ECD programs.

### Theme 3: Informal evaluation processes

The theme of Informal Evaluation Processes highlights the absence of structured monitoring and evaluation mechanisms in the programs. The data consistently revealed that monitoring relied heavily on occasional, unstructured visits from welfare officers and informal feedback, with no standardized procedures in place to assess the performance or outcomes of the programs. This lack of formalized evaluation protocols severely undermines the ability to systematically track progress, identify areas for improvement, or ensure accountability in the delivery of ECD services.

Occasional visits from welfare officers, but no structured evaluation.–ECD Teacher 1

This statement illustrates the sporadic nature of the evaluation process, indicating that welfare officers visit occasionally but do not adhere to any consistent or formalized plan. The teacher’s observation emphasizes that the reliance on these unstructured visits means that key aspects of the program’s effectiveness may go unaddressed, potentially leaving critical issues unresolved.

Occasional checks, no formal process.–Community Member 1

Similarly, a community member reinforces the theme of informality by acknowledging that evaluation is limited to occasional checks without any formalized structure. This lack of systematic evaluation processes likely leads to an incomplete assessment of the program’s effectiveness, as there is no comprehensive or regular review of key performance indicators. The absence of a formal evaluation framework limits the ability to understand the full scope of the program’s successes or failures.

Occasional subjective visits for monitoring.–Welfare Department Officer 1

The welfare officer’s quote confirms that not only are the visits occasional, but they are also subjective in nature. Without standardized criteria for evaluation, the monitoring process becomes heavily dependent on individual judgment. This can lead to inconsistent assessments, as different welfare officers may have varying perspectives on what constitutes success or areas for improvement. This subjectivity further undermines the potential for consistent and objective evaluation across different visits.

Limited monitoring, occasional visits.–Ministry of Education Officer 1

From the perspective of the Ministry of Education, the use of the word “limited” underscores the insufficient scope of the monitoring process. Monitoring is not only infrequent, but the occasional visits fail to provide a comprehensive evaluation of the program’s overall performance. The lack of structured follow-up or analysis limits the capacity to identify and address challenges systematically, reducing the program’s ability to improve in a meaningful way. Field observations further validated these findings, as there was no evidence of structured monitoring processes at any of the schools visited. Welfare officers and education officials made sporadic, informal visits with no clear follow-up or feedback loops in place. Community members, while actively involved in volunteer-driven aspects of the ECD programs, were not participating in any formal evaluation processes, reflecting a lack of accountability on both the governmental and community levels.

#### Impact of informal evaluation on ECD programs

The absence of structured evaluation processes presents significant challenges to the effectiveness of the monitoring and evaluation framework within ECD programs. Without formalized systems, it becomes difficult to ensure consistency, objectivity, and accountability. The informal nature of the evaluation process results in gaps in data collection, missed opportunities for program improvement, and an inability to consistently address areas of weakness. Furthermore, the reliance on occasional, subjective evaluations by individual welfare officers exacerbates these challenges, making it difficult to generate reliable and actionable insights.

The absence of consistent monitoring from both governmental bodies and the community aligns with the concerns raised in the interviews, highlighting a critical gap that limits the potential for quality improvements in ECD services. Without formal interventions or structured evaluation frameworks, it is unlikely that critical areas of performance will be systematically addressed. Establishing a more formalized evaluation system with regular monitoring and standardized criteria would significantly enhance the effectiveness of the evaluation process, ensuring that key performance indicators are consistently reviewed and improvements are made in a timely manner.

### Theme 4: Training and professional development

Participants consistently pointed to the irregularity and infrequency of training sessions, which were often constrained by limited resources and organizational challenges. Training opportunities appeared to lack consistency and were sporadically offered, leaving many participants inadequately equipped for their roles. This absence of regular and structured training likely hindered the professional development of ECD teachers and staff, as well as the overall effectiveness of the program.

As indicated by an ECD teacher,

Rarely attend formal training, occasional sessions organized by Social Welfare.

This quote reflects the infrequency of formal training opportunities. The use of "rarely" underscores the scarcity of such sessions, indicating that teachers receive little formal professional development, which can limit their ability to effectively perform their duties.

A similar sentiment was echoed by a community member:

Not actively involved in their training.

This observation highlights the disengagement and passivity in the training process. The lack of active involvement suggests that there is little emphasis on continuous development, further compounding the problem of inadequate training provisions.

The Welfare Department Officer further clarified:

We organize sporadic sessions due to funding limitations.

This statement reveals the root cause of the inconsistency—funding constraints. The sporadic nature of training stems not from a lack of intent but rather from practical limitations, such as financial resources. This resource scarcity leads to irregular training schedules, leaving staff without the necessary opportunities to build and refine their skills.

Finally, the Ministry of Education Official commented:

Occasional workshops, limitations due to resources.

which confirms the broader systemic issue. The occasional nature of the workshops, coupled with resource constraints, reflects a broader problem where structural and financial barriers impede the ability to offer regular, structured training sessions. The lack of consistent professional development opportunities not only affects the staff’s preparedness but also compromises the quality of education and care provided to the children.

Overall, the inconsistent and sporadic nature of training sessions is a direct result of resource limitations and organizational shortcomings. The irregularity of these training opportunities leaves staff underprepared and negatively impacts the effectiveness of the ECD program. Addressing these constraints through more structured and frequent training programs, coupled with securing adequate funding, would be essential steps in enhancing the program’s capacity to deliver high-quality education and care.

Building upon this theme, the findings from field notes and observations further corroborate the gaps identified in the thematic and sentiment analysis. Teachers frequently mentioned the lack of structured training opportunities in interviews, a sentiment reflected in the observation data, where no evidence of formal training programs or CPD initiatives was observed during the study period. Teachers were often seen relying on outdated or improvised teaching methods, further demonstrating the absence of skill development opportunities. Additionally, field notes revealed an apparent reliance on peer support and informal knowledge sharing among teachers, which, while useful, highlights the lack of institutionalized training structures. These observations reinforce the theme of inconsistent training and professional development, emphasizing the pressing need for more structured and frequent CPD initiatives to enhance the quality of ECD services.

### Theme 5: Support systems and professional development

The theme of Support Systems and Professional Development (CPD) further highlights the systemic challenges faced by ECD teachers, who are often left without adequate training or ongoing support once recruited. Both the interviews and observations underscore the absence of structured training programs and professional development opportunities, leaving teachers underprepared and unsupported in their roles. This issue is exacerbated by the fact that teachers often rely on outdated or informal methods of instruction with no consistent access to CPD.

We don’t get formal training. Once you’re in, you just learn as you go, mostly from other teachers or trial and error.–ECD Teacher 2

This quotation reflects the need for formal professional development pathways available to ECD teachers. With structured training, teachers are able to rely on peer support and self-teaching, which significantly hampers their ability to deliver high-quality education. The absence of CPD programs means that teachers are not exposed to modern teaching techniques or updates in ECD methodologies, further limiting their effectiveness in the classroom.

Field notes corroborated this issue, as teachers were observed relying on outdated or improvised materials and teaching methods. There needed to be evidence of regular training workshops or governmental initiatives to support the teachers in updating their skills. The observations revealed a reliance on traditional teaching techniques with slight variation or adaptation to newer pedagogical approaches. This lack of innovation in teaching methods was further attributed to the absence of CPD programs.

#### Volunteers’ perspective on lack of support

From the volunteers’ perspective, the absence of formal support systems and training opportunities was a major deterrent, contributing to their lack of long-term commitment to the role. Volunteers often expressed frustration with the lack of guidance and structured development, which left them feeling underqualified and unsupported.

We don’t get the kind of support that helps us improve. It feels like we’re just filling in gaps without any real backing from the authorities.–ECD Volunteer 1

This quote reflects the frustration of volunteers who, without professional support, feel they are merely “filling in” rather than developing as educators. The lack of structured training and clear career paths contributes to their dissatisfaction and eventual departure from their roles. This issue is further supported by research that shows that a lack of formal training and development opportunities results in high attrition rates among volunteers, as they are not given the tools to succeed and grow in their positions (Clarke, 2019).

#### Welfare officers’ lack of engagement in professional development.

When asked whether welfare officers were involved in facilitating or overseeing professional development programs, both teachers and officials noted that there was minimal engagement from welfare officers in supporting teacher growth after recruitment. Teachers essentially felt abandoned once recruited, with no ongoing evaluation or support from officials.

The welfare officers rarely come back after we’ve started teaching. There needs to be a follow-up to see how we’re doing or to provide any training.–ECD Teacher 4

This statement reveals the lack of continuity in welfare officers’ engagement with teachers after the recruitment phase. There is no structured system in place for welfare officers to monitor the teachers’ progress or ensure they receive professional development opportunities. This issue was further observed in the field, where no welfare officers conducted training sessions during the study period. Teachers were left without access to formal development opportunities or ongoing support, severely limiting their ability to improve their teaching methods and adapt to evolving educational demands.

The lack of support systems and professional development for ECD teachers is a critical issue affecting their ability to perform effectively. The absence of structured CPD programs, coupled with minimal involvement from welfare officers’ post-recruitment, leaves teachers without the tools or knowledge to grow professionally. The reliance on informal peer support and outdated teaching methods further exacerbates the challenges faced by ECD teachers, as they struggle to provide quality education without the necessary resources or guidance. The field observations reinforce these findings, revealing a clear gap in training and professional support, which ultimately leads to teacher burnout and high turnover rates. Addressing these systemic gaps is essential to improving the quality and sustainability of ECD programs in the region.

This thematic analysis reveals consistent patterns across participant groups, highlighting the lack of formal structures and standardized processes in ECD teachers’ recruitment, roles, evaluation, training, and support mechanisms. The reliance on community-driven initiatives and volunteerism significantly influences the dynamics within the early childhood education sector, underscoring challenges in establishing robust frameworks for effective ECD programs.

The sentiment analysis within the study offers a nuanced dissection of the diverse sentiments expressed by varied participant cohorts. By examining the sentiments of community members, educators, social welfare representatives, and government officials overseeing ECD, this analysis captures a broad spectrum of perspectives, revealing the complex landscape of perceptions regarding the effectiveness and challenges of ECD initiatives. This comprehensive scrutiny of both positive and negative sentiments elucidates the multifaceted experiences, concerns, and factors shaping stakeholders’ perceptions of ECD efforts. Table 2 illustrates the heterogeneity of sentiments across the participant groups.

**Table 2:** Descriptive statistics in sentiment mining.

Serial	Sentiments	Frequency %			
		Community members	ECD Teachers	Social welfare	Ministry officials
1	Positive	66	33	11	25
2	Negative	7	47	56	42
3	Neutral	27	20	33	33
4	Total	100	100	100	100

Table 2 presents a comparative analysis of positive and negative sentiments across each participant group. Community members exhibited a predominantly positive outlook towards the programs and processes, with 20 positive sentiments, only 3 negative sentiments, and 8 neutral sentiments. Conversely, teachers expressed higher negative sentiments, with 14 negative compared to 10 positive and 6 neutral sentiments. Similarly, social welfare members demonstrated significant negative sentiments, with 10 negative sentiments against 2 positive and 6 neutral sentiments. Ministry officials also reflected a negative sentiment bias, with 5 negative sentiments compared to 3 positive and 4 neutral sentiments.

## Discussion

The study investigates the paradigms of recruitment, role elucidation, and support mechanisms within the domain of ECD in T/A Zilakoma, Nkhata Bay South Constituency in Malawi, employing a thematic analysis as a methodological approach. In tandem with this objective, the exploration derives insights from interviews conducted among diverse stakeholders, including ECD educators, community members, welfare officers, and Ministry of Education officials.

The thematic outcomes gleaned from these interviews, delving into recruitment practices, role ambiguity, monitoring and evaluation protocols, training endeavors, and the dependency on community structures, coalesce prominently with Bronfenbrenner’s theory. The resonance between the theoretical underpinning and the derived themes elucidates the intricate interplay between various contextual layers influencing ECD settings.

The discerned thematic elements, interwoven with the study objectives, present a comprehensive portrait of the prevalent challenges and dynamics within ECD environments. The EST, conceptualizing the nested systems shaping human development, resonates deeply with the observed nuances in recruitment procedures marked by community-centric endorsements, ambiguous role delineation impacting educators, informal monitoring practices, sporadic training opportunities, and the pronounced reliance on community-driven initiatives.

This alignment between the study’s objectives and the thematic revelations derived from participant narratives accentuates the intricate interconnections within the educational ecosystem. The thematic analysis, when viewed through the lens of the EST, offers a rich understanding of the multilayered influences pervading the ECD landscape, shedding light on the systemic challenges and informalities entrenched within critical facets of early childhood education.

In essence, the thematic analysis not only amplifies the study objectives but also harmonizes intricately with the foundational premises of the EST, unveiling the nuanced tapestry of influences shaping the landscape of early childhood education.

The thematic analysis conducted within this study offers a comprehensive exploration into the perceptions and experiences of stakeholders engaged in early childhood education, specifically focusing on recruitment, roles, and support mechanisms, corroborating extant research. It should be borne in mind at this point that recruitment of unqualified human resources not only infringes on child rights to access quality education, but also presents a serious danger to a child’s health and subjugates underprivileged children to adverse social challenges such as discrimination against the special needs children [16].

The first discerned theme, "Recruitment Practices," delineates a landscape characterized by a lack of formalized structures prevailing across participant groups. These findings corroborate extant studies within Sub-Saharan Africa [5,17,42]. The narratives from various stakeholders vividly highlight an absence of systematic recruitment processes, with reliance predominantly placed upon community recommendations and voluntary engagements. This pattern is succinctly encapsulated in participant quotations, underscoring the essence of community-driven recruitment practices, wherein personal recommendations and volunteerism supersede formal frameworks. This theme illuminates the prevalent informality and community-centric nature ingrained within the recruitment milieu. Such recruitment techniques are devoid of clear guidelines and technical qualifications, leading to the recruits having no clear TORs.

Within the thematic domain of "Role Ambiguity (Terms of Reference—TORs)," the sub-theme "Undefined Responsibilities" surfaces prominently, portraying a pervasive sense of ambiguity surrounding the delineation of duties and responsibilities incumbent upon ECD teachers. Across participant groups, a consensus emerges regarding the lack of clearly defined TORs, leading to an operational environment where duties are perceived as flexible and molded by individual willingness rather than structured guidelines. This overarching lack of specificity accentuates the unstructured nature of role expectations within the context of early childhood education and poses a significant threat to the efficiency of ECD facilities [16].

The findings from this study reveal a critical issue in the ECD: The teachers often do not remain in their positions for extended periods, likely due to dissatisfaction with salaries and unfavorable working conditions. This high turnover rate presents a significant challenge to the sustainability and quality of ECD services. The frustration experienced by teachers, driven by inadequate financial compensation and poor working conditions, is compounded by the lack of formal training in ECD and the absence of a clear career path, leading many to abandon the profession entirely.

Research corroborates these findings, illustrating that poor working conditions, including low wages, are significant contributors to global teacher attrition in ECD settings. According to Clarke (2019), low wages and the absence of job security are primary factors that lead to the high turnover of ECD educators, particularly in rural and underfunded areas. In contexts where ECD services are community-driven and volunteer-based, as seen in this study, the lack of formalized roles and structured career progression further demotivates teachers. This issue is mirrored in studies from Sub-Saharan Africa, where the recruitment of unqualified and poorly compensated teachers negatively impacts not only their retention but also the quality of care and education provided to children [29].

Moreover, the absence of professional development opportunities exacerbates the situation. Teachers often find themselves in roles with undefined expectations and no opportunity for career advancement, which creates a sense of stagnation. Research has shown that access to continuous professional development is crucial in enhancing teacher motivation and retention. Davis (2018) argues that structured training programs not only improve the skills and competence of ECD educators but also foster a sense of professional identity and purpose, which are crucial to reducing turnover. The lack of such opportunities in T/A Zilakoma contributes to many ECD teachers’ frustration and eventual departure.

Moreover, the theme "Training and Professional Development" unveils the sub-theme "Inconsistent Training Opportunities," highlighting the sporadic nature of training sessions available to ECD teachers. Constrained by resource limitations, participants express a shared sentiment regarding the infrequency and inadequacy of formal training, primarily organized sporadically due to financial constraints. This depiction underscores the challenges encountered in fostering consistent and robust professional development opportunities within the ECD domain and is consistent with findings by [16]. Additionally, the failure to establish a clear career path for ECD educators undermines the professionalization of the sector. Fenech et al. (2009) highlight that in settings where early childhood educators are viewed merely as caregivers rather than professionals, there is little incentive for teachers to remain in the field. Without precise career trajectories or pathways for advancement, teachers feel undervalued and are more likely to leave the profession. This is particularly problematic in rural areas, where resources and support are limited, and the professional standing of ECD educators is further marginalized.

The high turnover of ECD teachers, driven by these systemic issues, has profound implications for the quality of early childhood education. Consistency and stability in teacher-child relationships are crucial for young children’s cognitive and socio-emotional development, as noted by Yunus et al. (2023). Frequent changes in staff disrupt these relationships, undermining the effectiveness of ECD programs and potentially compromising children’s developmental outcomes. This corroborates extant studies highlighting that teacher turnover in under-resourced settings can lead to a cycle of poor quality, where the lack of trained and committed educators perpetuates suboptimal learning environments [23].

Furthermore, the theme "Monitoring and Evaluation Practices" unravels the "Informal Evaluation Processes" sub-theme, spotlighting the absence of structured assessment mechanisms. The consensus among participants across groups revolves around sporadic and subjective evaluation practices, primarily relying on occasional visits and informal feedback loops. This portrayal accentuates the prevailing dearth of formalized evaluation protocols, shaping an environment characterized by limited and inconsistent monitoring practices.

Lastly, the theme "Dependency on Community and Volunteerism" delineates a sub-theme labeled "Community-Driven Approach," emblematic of the overarching reliance on community recommendations and volunteerism shaping recruitment and support structures. Participants uniformly underscore the pivotal role played by community needs and recommendations, underscoring a recruitment ethos primarily governed by community-driven initiatives, devoid of formalized structures, contrary to principles of specialization and clearly outlined roles as demanded in formal organizations [15]. Much as volunteerism is associated with intrinsic motivation, it may pose the dangers of incompetency.

In summation, this thematic analysis serves as a revelatory lens into the intricate dynamics of recruitment, role definition, monitoring practices, training paradigms, and community-driven mechanisms within the realm of early childhood education. The divergent perspectives and recurring themes extracted from stakeholder narratives present a nuanced portrayal of the multifaceted landscape characterizing the domain, highlighting prevailing challenges and informalities entrenched within these pivotal aspects of early childhood education.

The disparity in sentiment among different participant groups within the study could be indicative of various factors influencing their perspectives on the ECD programs and processes.

The overwhelmingly positive sentiment among community members suggests a supportive stance toward the ECD programs. This positivity might stem from their direct observation of the benefits these programs bring to their communities. They may appreciate the educational opportunities provided for their children and the overall positive impact on their locality.

The higher incidence of negative sentiments among teachers could highlight underlying issues they face within the ECD programs. Possible reasons might include inadequate resources, limited professional development opportunities, challenges in managing diverse classrooms, or a lack of structured support from authorities. Negative sentiments could also stem from frustration due to unaddressed concerns or the overwhelming responsibilities they bear without adequate support.

Like teachers, negative sentiments among social welfare members might arise from the challenges they encounter in overseeing and managing ECD programs. Issues such as insufficient funding, logistical hurdles, or difficulties ensuring quality standards might contribute to their negative perceptions. Limited resources and a sense of inadequacy in fulfilling their responsibilities might drive their negative sentiments.

The relatively higher occurrence of negative sentiments among ministry officials could indicate their awareness of systemic challenges within the programs. These officials might grapple with bureaucratic limitations, budget constraints, or policy gaps that hinder effective implementation. The negative sentiments could be reflective of their frustrations in navigating administrative hurdles or witnessing shortcomings in achieving program objectives.

In summary, the disparity in sentiments reflects the varying experiences and challenges encountered by different stakeholders involved in the programs. Positive sentiments from community members might contrast with the more negative perspectives of teachers, social welfare members, and ministry officials due to their distinct vantage points and the diverse challenges they confront within the ECD landscape.

## Conclusion

In conclusion, this study provides an in-depth examination of the challenges and opportunities within the ECD landscape in Malawi, with a specific focus on rural Community-Based Childcare Centres (CBCCs). Grounded in a robust theoretical framework that integrates socio-cultural and educational perspectives, the research identifies critical gaps in the current ECD system, such as the absence of structured recruitment processes, undefined roles for educators, and inadequate support and training mechanisms. These deficiencies not only limit the quality and effectiveness of ECD services but also hinder their scalability and sustainability throughout the country. The study highlights substantial obstacles in policy implementation, resource allocation, and access to quality ECD, particularly in remote areas. These issues are compounded by a lack of infrastructure, a shortage of qualified educators, and inconsistent program delivery, further exacerbated by financial constraints. Consequently, the research underscores the need for a systematic approach to strengthening ECD frameworks by integrating evidence-based best practices known to be effective in similar global contexts.

### Implications for policy

The findings of this study have several important policy implications. First, there is an urgent need for policy reforms that formalize recruitment and training processes to professionalize ECD practice across Malawi. Establishing standardized recruitment procedures and providing clear role definitions for educators will foster a more structured environment, enabling educators to deliver more effective education. Additionally, ongoing professional development tailored to the specific needs of ECD educators is essential for improving the quality of education and care provided to young children.

Second, the study emphasizes the importance of equitable resource allocation, particularly for rural areas, where the shortage of qualified teachers and inadequate infrastructure are most acute. Policies should focus on improving funding for ECD centers to address these disparities and ensure that children in all regions have access to quality early childhood education.

Third, collaborative efforts between government officials, local communities, and international partners are necessary to tailor ECD interventions that meet local needs while aligning with global standards. These collaborative efforts should include the creation of robust monitoring and evaluation mechanisms to ensure consistent quality across ECD centers. By addressing these policy challenges, Malawi can create a more sustainable and effective ECD system that supports the development of its youngest learners, setting a foundation for future socio-economic growth.

### Study limitations and contributions

One limitation of this study is the sample size, which may limit the generalizability of the findings beyond the specific region studied, T/A Zilakoma. While the insights gathered are valuable for understanding the local context, they may not fully capture the broader experiences of ECD practices across different regions. The reliance on a relatively small group of participants, including teachers and officials from one constituency, means that regional variations and other contextual factors could influence the applicability of the results to other areas. Additionally, the study’s dependence on self-reported data through interviews presents potential bias, as participants may unintentionally overstate or understate aspects of their experiences, influenced by memory, social desirability, or personal perception.

### Contributions of this study

The multifaceted contributions of this study can be viewed through several dimensions:

Contextual Insight: The study provides a detailed examination of the ECD landscape within a rural Malawian context, specifically in T/A Zilakoma. This localized focus offers insight into the challenges of rural-based ECD centers, adding to the existing literature by offering region-specific data and analysis, often lacking in the broader ECD discourse.

Policy and Practice Recommendations: By identifying key challenges in recruitment practices, role clarity, training, and reliance on volunteerism, the study proposes actionable policy recommendations. These are particularly valuable for stakeholders seeking to professionalize the ECD sector, offering clear guidelines for improving recruitment, monitoring, and training systems within the sector.

Theoretical Application: The application of the EST provides a nuanced analysis of how various environmental systems (micro, meso, exo, macro, and chrono) interact to influence ECD outcomes. This adds a theoretical richness to the study, providing a structured lens through which to view the multifaceted challenges within the ECD framework.

Contribution to Global Discussions: Despite its focus on a rural Malawian setting, the study’s findings have broader implications for other developing countries facing similar ECD challenges. This comparative relevance contributes to the global discourse on achieving equitable and quality early childhood education in resource-constrained environments.

Sentiment Analysis as a Novel Approach: The inclusion of sentiment analysis adds an innovative layer to understanding stakeholders’ experiences and perceptions. This methodological approach provides a broader view of how different actors perceive the effectiveness of ECD programs, offering a more profound understanding of community, educator, and governmental responses to ECD initiatives.

### Future study

To address these limitations, future research could expand the sample size to include a broader range of participants from various regions, enhancing the generalizability of the findings. Furthermore, complementing interviews with quantitative methods or observational data could provide a more robust and objective analysis of ECD practices, reducing the impact of self-report bias and offering a more comprehensive understanding of the dynamics in early childhood education.

## Supporting information

S1 Data (ZIP)
