# Supplementary material for: Challenges and realities of early childhood development centers in Malawi: A critical examination
Source: PLoS One. 2025 Feb 21;20(2):e0314530. doi: 10.1371/journal.pone.0314530 (PMC11844827; doi:10.1371/journal.pone.0314530)
Supplement: S1 Data — (ZIP) [file pone.0314530.s001.zip › ECD Teacher 3.docx]

ECD Teacher 3:

*What are the primary challenges you face in delivering ECD services?*

The biggest challenge is the lack of professionally trained staff. Without proper training, we struggle to maintain a high standard of teaching. There's also a complete absence of CPDs, which hinders our professional growth. The physical setting of our teaching spaces, mostly in churches, is not suitable for young children. Many parents are unable to pay the fees, affecting our operations and leading to lower enrollment. A lack of understanding of ECD's importance amongst some parents also poses a challenge. Furthermore, the issue of accessibility is significant, as some children have to travel long distances without proper transportation means.
