# Supplementary material for: Challenges and realities of early childhood development centers in Malawi: A critical examination
Source: PLoS One. 2025 Feb 21;20(2):e0314530. doi: 10.1371/journal.pone.0314530 (PMC11844827; doi:10.1371/journal.pone.0314530)
Supplement: S1 Data — (ZIP) [file pone.0314530.s001.zip › PARENT 6.docx]

ECD INTERVIEWS WITH PARENTS

Parent 2: "

1. ***Distance***

The problem is that we are far from the ECD centers. It’s tiring to walk, taking the kid there and back everyday.

1. ***Teacher quality***

"Our ECD center's staff lacks formal training. I'm worried it's affecting the quality of my child's early education."

1. ***Financial***

"The ECD fees are a burden for us. It's becoming increasingly difficult to keep up with the payments."

1. ***infrastructure***

"The center operates in a building, with adult-sized chairs. It's not an ideal learning environment for young children."
