# Supplementary material for: Challenges and realities of early childhood development centers in Malawi: A critical examination
Source: PLoS One. 2025 Feb 21;20(2):e0314530. doi: 10.1371/journal.pone.0314530 (PMC11844827; doi:10.1371/journal.pone.0314530)
Supplement: S1 Data — (ZIP) [file pone.0314530.s001.zip › Welfare Department Officer 1.docx]

Welfare Department Officer 1:

Recruitment: "Accept volunteers or community recommendations without structure."

TORs: "No defined TORs, responsibilities based on willingness."

Evaluation: "Occasional subjective visits for monitoring."

Training: "We organize sporadic sessions due to funding limitations."

Monitoring: "Occasional visits, lacks a standardized process."
