# Supplementary material for: Challenges and realities of early childhood development centers in Malawi: A critical examination
Source: PLoS One. 2025 Feb 21;20(2):e0314530. doi: 10.1371/journal.pone.0314530 (PMC11844827; doi:10.1371/journal.pone.0314530)
Supplement: S1 Data — (ZIP) [file pone.0314530.s001.zip › ECD Teacher 4.docx]

Teacher 4:

Recruitment: "Community-driven, volunteered to teach."

TORs: "No specific duties, focused on childcare and teaching."

Evaluation: "Informal feedback, no formal evaluation process."

Updates: "Limited exposure to training sessions."

Monitoring: "Occasional visits from welfare officers, no formal process."
