# Supplementary material for: Challenges and realities of early childhood development centers in Malawi: A critical examination
Source: PLoS One. 2025 Feb 21;20(2):e0314530. doi: 10.1371/journal.pone.0314530 (PMC11844827; doi:10.1371/journal.pone.0314530)
Supplement: S1 Data — (ZIP) [file pone.0314530.s001.zip › ECD Teacher 5.docx]

ECD Teacher 5:

*What are the impacts of these challenges on ECD services?*

The impacts are far-reaching. Without trained teachers and suitable learning environments, the quality of education we provide is compromised. We're also unable to engage in ongoing learning and development as educators, which is crucial in the ECD field. The physical setup of our centers, often in buildings designed elders, with adult-sized furniture, is not conducive to young children's learning.
