# Supplementary material for: Challenges and realities of early childhood development centers in Malawi: A critical examination
Source: PLoS One. 2025 Feb 21;20(2):e0314530. doi: 10.1371/journal.pone.0314530 (PMC11844827; doi:10.1371/journal.pone.0314530)
Supplement: S1 Data — (ZIP) [file pone.0314530.s001.zip › ECD Teacher 6.docx]

ECD Teacher 6:

*What improvements would you like to see in the ECD sector?*

We desperately need professional training and opportunities for continuous development. This would greatly enhance the quality of education we can offer. Improved infrastructure, with child-friendly facilities, is also crucial. Addressing the affordability issue is important – perhaps through subsidies or a sliding scale fee system. Increasing awareness among parents about the importance of ECD is also essential. And finally, making ECD centers more accessible, especially for children from remote areas, would go a long way in improving enrollment and retention.
