# Supplementary material for: Challenges and realities of early childhood development centers in Malawi: A critical examination
Source: PLoS One. 2025 Feb 21;20(2):e0314530. doi: 10.1371/journal.pone.0314530 (PMC11844827; doi:10.1371/journal.pone.0314530)
Supplement: S1 Data — (ZIP) [file pone.0314530.s001.zip › ECD Teacher 7.docx]

ECD Teacher 7:

*Can you elaborate on the challenges related to parental perception of ECD?*

Sure. There's a noticeable divide in parental attitudes towards ECD. While some parents are very supportive and understand the importance of early education, others don't see its value. This dichotomy affects enrollment and engagement levels. Parents who don't perceive the importance of ECD often choose not to enroll their children, missing out on the crucial benefits of early learning.
