# Supplementary material for: Challenges and realities of early childhood development centers in Malawi: A critical examination
Source: PLoS One. 2025 Feb 21;20(2):e0314530. doi: 10.1371/journal.pone.0314530 (PMC11844827; doi:10.1371/journal.pone.0314530)
Supplement: S1 Data — (ZIP) [file pone.0314530.s001.zip › ECD Teacher 9 copy.docx]

ECD Teacher 9:

*What would be the ideal scenario for ECD here?*

Ideally, we would have a team of trained ECD professionals equipped with the latest educational techniques. Continuous professional development would keep us updated and improve our teaching quality. Child-friendly, purpose-built ECD centers would provide a conducive learning environment. If ECD were more affordable, or even free, it would increase accessibility for all children. Raising awareness among all parents about the importance of ECD would also boost enrollment. And, of course, addressing the transportation issue for remote learners is crucial.
