# Supplementary material for: Challenges and realities of early childhood development centers in Malawi: A critical examination
Source: PLoS One. 2025 Feb 21;20(2):e0314530. doi: 10.1371/journal.pone.0314530 (PMC11844827; doi:10.1371/journal.pone.0314530)
Supplement: S1 Data — (ZIP) [file pone.0314530.s001.zip › ECD Teacher 10.docx]

Teacher 4:

*How do you manage with the existing challenges in providing ECD education?*

It's a constant struggle. Our lack of formal training in early childhood education severely limits our ability to provide quality services. The absence of continuous professional development means we're not keeping up with the latest teaching methods. Additionally, the teaching spaces are far from ideal – they're not designed for young learners, and some are just rudimentary shelters. Many parents can't afford the fees, leading to low retention rates. Also, there's a significant divide among parents; while some are very passionate about ECD, others don't see its value, which affects enrollment. Lastly, accessibility is a major issue for children living far away without reliable transportation.

Financial constraints for families lead to inconsistent attendance. Moreover, the lack of awareness among some parents about the benefits of ECD hinders children's participation. And for those living far from the centers, the lack of transport poses a significant barrier.
