# Supplementary material for: Challenges and realities of early childhood development centers in Malawi: A critical examination
Source: PLoS One. 2025 Feb 21;20(2):e0314530. doi: 10.1371/journal.pone.0314530 (PMC11844827; doi:10.1371/journal.pone.0314530)
Supplement: S1 Data — (ZIP) [file pone.0314530.s001.zip › ECD Teacher interviews.docx]

The following are interviews with ECD teachers

ECD Teacher 1:

*Could you share your insights on the quality and accessibility of ECD services in here?*

Certainly. We're facing a serious challenge due to the lack of trained teachers, which directly impacts the quality of education we can provide. There are no continuous professional development opportunities, leaving us with outdated teaching methods. The facilities we use, often churches, aren’t child-friendly – the seating is designed for adults, and some centers are just simple structures, hardly protecting the kids from harsh weather. Many parents struggle to afford the fees, leading to low retention rates. Also, there's a general lack of awareness among some parents about the importance of ECD, which affects enrollment. Additionally, accessibility is a major issue as some children live too far away and have no means of transport.

ECD Teacher 2:

*Can you describe your experience as an ECD teacher in here?*

Teaching here is challenging. We lack proper training, which makes it difficult to provide quality education. The classrooms aren’t designed for young children – we often use church buildings with adult-sized chairs. This environment is not conducive to early learning. On top of that, many parents can't afford the fees, resulting in low retention. Some parents don't see the importance of early education. Moreover, for some children, the distance to the center is a major barrier, as they don’t have any means of transport.

ECD Teacher 3:

*What are the primary challenges you face in delivering ECD services?*

The biggest challenge is the lack of professionally trained staff. Without proper training, we struggle to maintain a high standard of teaching. There's also a complete absence of CPDs, which hinders our professional growth. The physical setting of our teaching spaces, mostly in churches, is not suitable for young children. Many parents are unable to pay the fees, affecting our operations and leading to lower enrollment. A lack of understanding of ECD's importance amongst some parents also poses a challenge. Furthermore, the issue of accessibility is significant, as some children have to travel long distances without proper transportation means.

Teacher 4:

Recruitment: "Community-driven, volunteered to teach."

TORs: "No specific duties, focused on childcare and teaching."

Evaluation: "Informal feedback, no formal evaluation process."

Updates: "Limited exposure to training sessions."

Monitoring: "Occasional visits from welfare officers, no formal process."

ECD Teacher 5:

*What are the impacts of these challenges on ECD services?*

The impacts are far-reaching. Without trained teachers and suitable learning environments, the quality of education we provide is compromised. We're also unable to engage in ongoing learning and development as educators, which is crucial in the ECD field. The physical setup of our centers, often in churches with adult-sized furniture, is not conducive to young children's learning.

ECD Teacher 6:

*What improvements would you like to see in the ECD sector?*

We desperately need professional training and opportunities for continuous development. This would greatly enhance the quality of education we can offer. Improved infrastructure, with child-friendly facilities, is also crucial. Addressing the affordability issue is important – perhaps through subsidies or a sliding scale fee system. Increasing awareness among parents about the importance of ECD is also essential. And finally, making ECD centers more accessible, especially for children from remote areas, would go a long way in improving enrollment and retention.

ECD Teacher 7:

*Can you elaborate on the challenges related to parental perception of ECD?*

Sure. There's a noticeable divide in parental attitudes towards ECD. While some parents are very supportive and understand the importance of early education, others don't see its value. This dichotomy affects enrollment and engagement levels. Parents who don't perceive the importance of ECD often choose not to enroll their children, missing out on the crucial benefits of early learning.

ECD Teacher 8:

*How do you cope with the challenges of providing ECD in such conditions?*

It's challenging, but we do our best. The lack of trained professionals is a significant issue. We often rely on our instincts rather than formal teaching methods. The absence of CPDs means we're not able to update our skills. Facilities are another major issue – using spaces like churches is far from ideal for young children. The financial burden on parents leads to low attendance, and the varying levels of awareness about ECD's importance further complicate the situation. Transport issues for children living far from the centers also hinder access to education.

ECD Teacher 9:

*What would be the ideal scenario for ECD in here?*

Ideally, we would have a team of trained ECD professionals equipped with the latest educational techniques. Continuous professional development would keep us updated and improve our teaching quality. Child-friendly, purpose-built ECD centers would provide a conducive learning environment. If ECD were more affordable, or even free, it would increase accessibility for all children. Raising awareness among all parents about the importance of ECD would also boost enrollment. And, of course, addressing the transportation issue for remote learners is crucial.

Teacher 10

*How do you manage with the existing challenges in providing ECD education?*

It's a constant struggle. Our lack of formal training in early childhood education severely limits our ability to provide quality services. The absence of continuous professional development means we're not keeping up with the latest teaching methods. Additionally, the teaching spaces are far from ideal – they're not designed for young learners, and some are just rudimentary shelters. Many parents can't afford the fees, leading to low retention rates. Also, there's a significant divide among parents; while some are very passionate about ECD, others don't see its value, which affects enrollment. Lastly, accessibility is a major issue for children living far away without reliable transportation.

Financial constraints for families lead to inconsistent attendance. Moreover, the lack of awareness among some parents about the benefits of ECD hinders children's participation. And for those living far from the centers, the lack of transport poses a significant barrier.
