# Supplementary material for: Challenges and realities of early childhood development centers in Malawi: A critical examination
Source: PLoS One. 2025 Feb 21;20(2):e0314530. doi: 10.1371/journal.pone.0314530 (PMC11844827; doi:10.1371/journal.pone.0314530)
Supplement: S1 Data — (ZIP) [file pone.0314530.s001.zip › Ministry 1.docx]

Ministry of Education Official 1:

*How does the Ministry of Education view its role in supporting ECD in Malawi?*

Our role is crucial. We are responsible for providing guidance and support to ECD programs. However, we face significant challenges, particularly in ensuring the effective implementation of our policies. There's a gap between policy and practice, primarily due to limited monitoring and resources.

*What are the main challenges in aligning policy with actual implementation?*

The biggest challenge is resource allocation. Our policies emphasize quality and accessibility in ECD, but we struggle with limited funding and human resources. Additionally, there's a lack of comprehensive monitoring, which hinders our ability to evaluate and adjust programs effectively.

*What improvements would be necessary to address these issues?*

Strengthening our monitoring systems is key. We also need to secure more funding and improve our coordination with various stakeholders involved in ECD. Providing continuous professional development for teachers is another critical area we need to focus on.
