# Supplementary material for: Challenges and realities of early childhood development centers in Malawi: A critical examination
Source: PLoS One. 2025 Feb 21;20(2):e0314530. doi: 10.1371/journal.pone.0314530 (PMC11844827; doi:10.1371/journal.pone.0314530)
Supplement: S1 Data — (ZIP) [file pone.0314530.s001.zip › Ministry 2.docx]

Ministry of Education Official 2:

*Can you discuss the Ministry's role in ensuring quality ECD services?*

Absolutely. Our role involves setting standards and overseeing the implementation of ECD services. However, we acknowledge that there is a disconnection between our policy frameworks and the realities on the ground. This is largely due to insufficient monitoring and a lack of resources.

*What specific challenges do you face in implementing ECD policies?*

One of the major challenges is the discrepancy between our policies and the actual conditions in ECD centers, especially in terms of infrastructure and teacher training. Many ECD centers lack proper facilities, and there is a significant need for trained educators.

*What steps do you believe are necessary to bridge these gaps?*

We need to increase investment in ECD, both in terms of physical infrastructure and human resources. It's also vital that we develop and maintain a rigorous system for monitoring and evaluating ECD programs to ensure they meet the set standards. Additionally, enhancing collaboration with other ministries and NGOs is crucial for a more holistic approach to ECD.
