# Supplementary material for: Challenges and realities of early childhood development centers in Malawi: A critical examination
Source: PLoS One. 2025 Feb 21;20(2):e0314530. doi: 10.1371/journal.pone.0314530 (PMC11844827; doi:10.1371/journal.pone.0314530)
Supplement: S1 Data — (ZIP) [file pone.0314530.s001.zip › NGO Official 1.docx]

Interview with NGO Official 1:

*What is your organization's role in supporting ECD in Malawi?*

Our NGO works to complement the government's efforts in ECD by providing resources, training, and support to local ECD centers. However, we often encounter challenges due to the lack of coordination with government agencies and other NGOs. This sometimes leads to overlapping efforts and inefficient use of resources.

*How do you perceive the current state of ECD services in Malawi?*

While there are many dedicated individuals working in ECD, the overall system faces significant issues, especially in terms of quality and accessibility. Many ECD centers lack adequate facilities, and there's a dire need for trained teachers. Additionally, community awareness about the importance of ECD is still quite low.

*What improvements would you suggest?*

First, there needs to be better coordination between the government, NGOs, and other stakeholders in ECD. Also, more investment is needed, not just in terms of funding, but also in building the capacities of ECD providers. We also advocate for stronger community engagement to raise awareness about the value of early childhood education.
