# Supplementary material for: Challenges and realities of early childhood development centers in Malawi: A critical examination
Source: PLoS One. 2025 Feb 21;20(2):e0314530. doi: 10.1371/journal.pone.0314530 (PMC11844827; doi:10.1371/journal.pone.0314530)
Supplement: S1 Data — (ZIP) [file pone.0314530.s001.zip › Social Welfare Officer 2.docx]

Interview with Social Welfare Officer 2:

*What is your perspective on the current state of ECD in Malawi?*

The current state is challenging. Our role as social welfare officers is to collaborate with ECD implementers, but the lack of a strong, centralized directive from higher authorities like the Ministry makes our task difficult. The absence of a clear mandate and sufficient resources significantly reduces our effectiveness in the field.

*What are the main barriers to effective ECD implementation?*

The fragmentation of efforts among ECD stakeholders, including ourselves, NGOs, and community organizations, is a significant barrier. The lack of coordination results in some regions receiving more attention than others. It’s a cycle of uneven resource distribution and service provision.

*What improvements would you suggest?*

We need a more cohesive strategy for ECD implementation. This includes better coordination and communication among all stakeholders and a centralized funding mechanism. If we could streamline our efforts under a common goal and better distribute resources, we could significantly enhance the reach and quality of ECD services.
